# Supplementary material for: Graph-based pan-genome reveals structural and sequence variations related to agronomic traits and domestication in cucumber
Source: Nat Commun. 2022 Feb 3;13:682. doi: 10.1038/s41467-022-28362-0 (PMC8813957; doi:10.1038/s41467-022-28362-0)
Supplement: Supplementary file 1 — Supplementary Information [file 41467_2022_28362_MOESM1_ESM.pdf]

Graph-based pan-genome reveals structural and sequence variations related to agronomic traits and domestication in cucumber

Li *et al.*

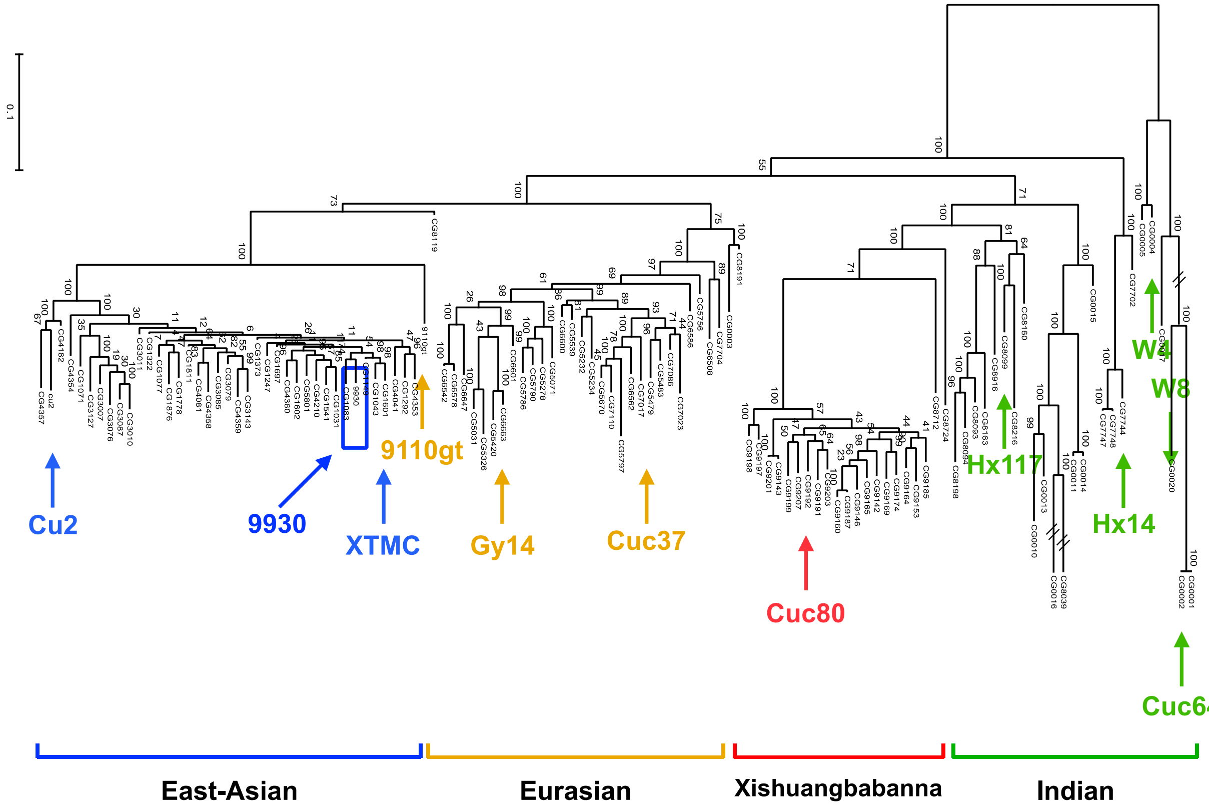

Supplementary Fig. 1. Positions of the 12 cucumber accessions used in this study on the phylogenetic tree. The tree of 115 cucumber accessions is modified from our previous study[1]. Source data are provided as a Source Data file.

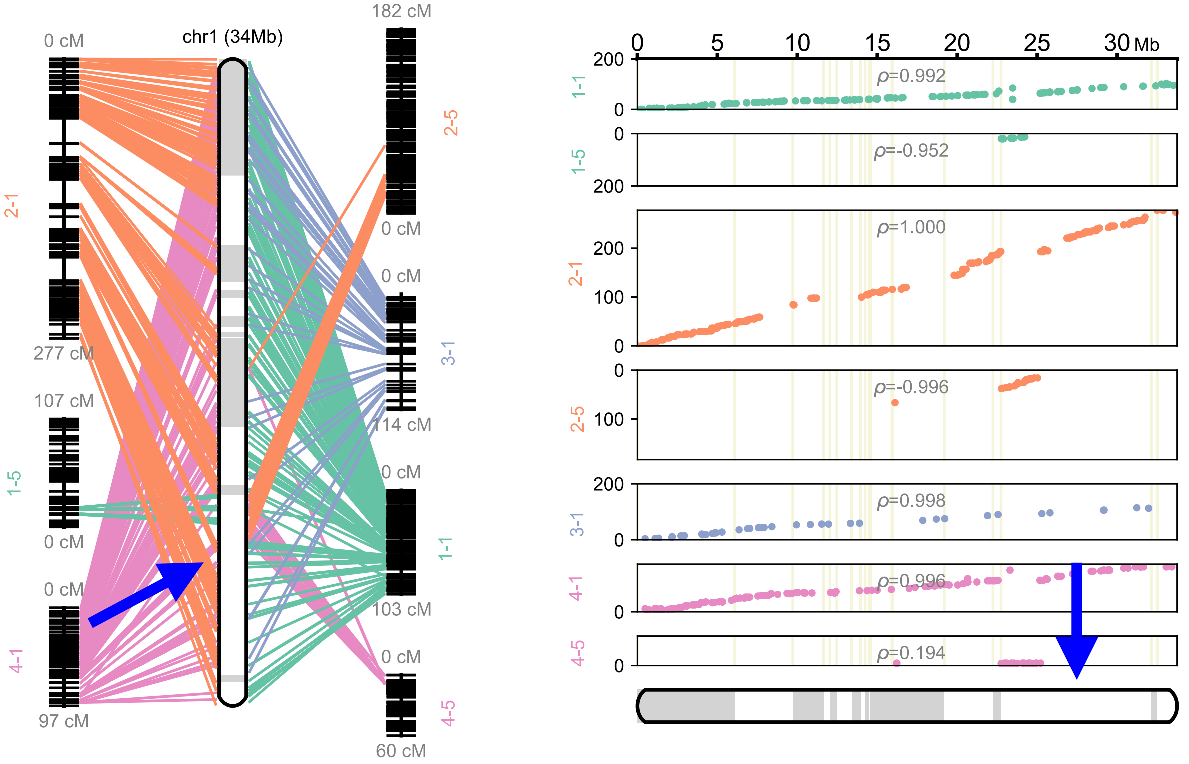

Supplementary Fig. 2. An example of splitting a misassembled contig (tig00002967_pilon) of Cuc64 based on four linkage maps. Blue arrow indicates the problematic contig.

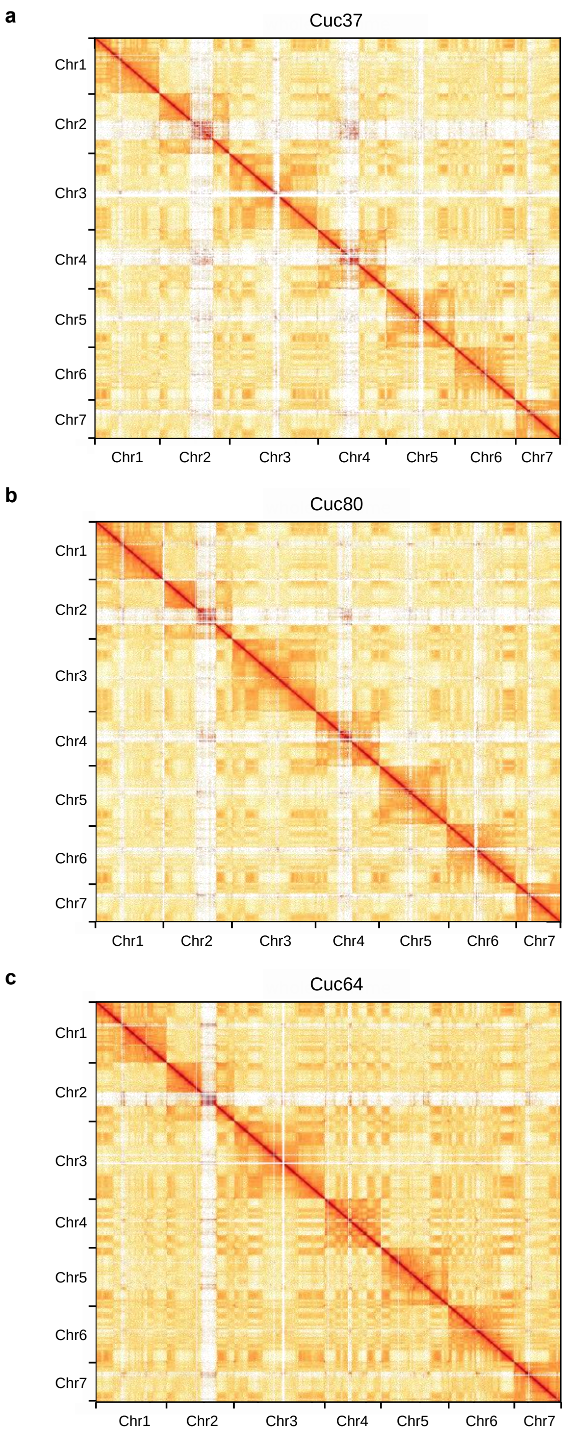

Supplementary Fig. 3. Hi-C interaction heatmaps at the 100-kb resolution. a, Hi-C heatmap of the Cuc37 genome. **b,** Hi-C heatmap of the Cuc80 genome. **c,** Hi-C heatmap of the Cuc64 genome. Red color indicates strong interactions.

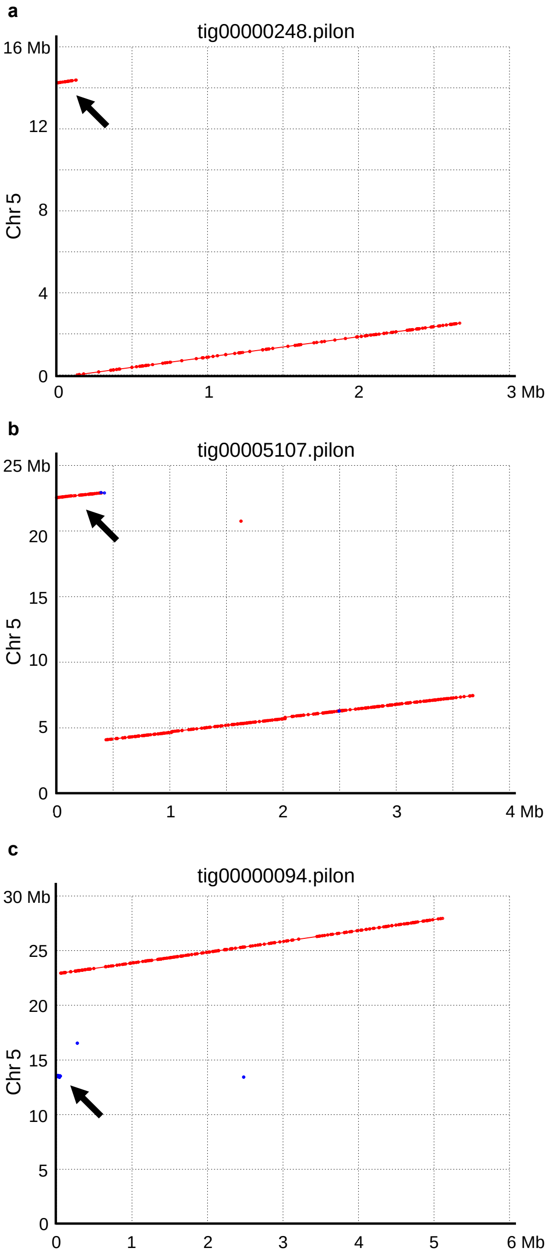

Supplementary Fig. 4. Breakpoint evidence of the three large inversions on chromosome 5 between W8 and 9930. a-c, Alignments of three assembled contigs of W8 harboring inversion breakpoints, tig00000248.pilon (**a**), tig00005107.pilon (**b**) and tig00005107.pilon (**c**) (x-axis) to chromosome 5 of 9930 (y-axis). Black arrows denote the positions of breakpoints.

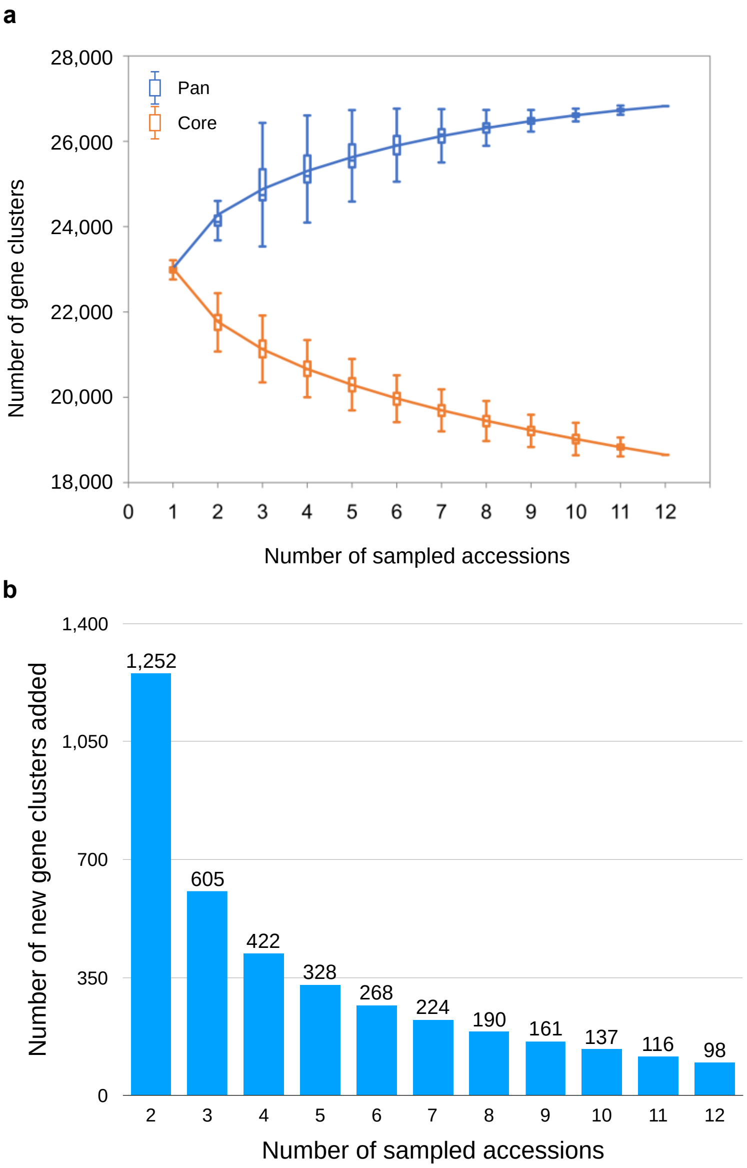

Supplementary Fig. 5. **Simulation of pan-genome and core genome sizes and newly added gene clusters. a,** Pan-genome and core genome size simulation in terms of the number of gene clusters. The median and upper and lower quartiles of gene cluster numbers are shown in boxplots. The whiskers extend to the minimum and maximum of the data. The pan- and core-genome curves were fitted using nonlinear models recommended by Tettelin *et al.*[2]. Simulation of pan-genome size in terms of the number of protein-coding genes was performed with PanGP[3] using the totally random algorithm with the number of combinations at each given number of genomes set to 500 and the sample replication time set to 20. **b,** Number of newly added gene clusters when including more accessions. Source data are provided as a Source Data file.

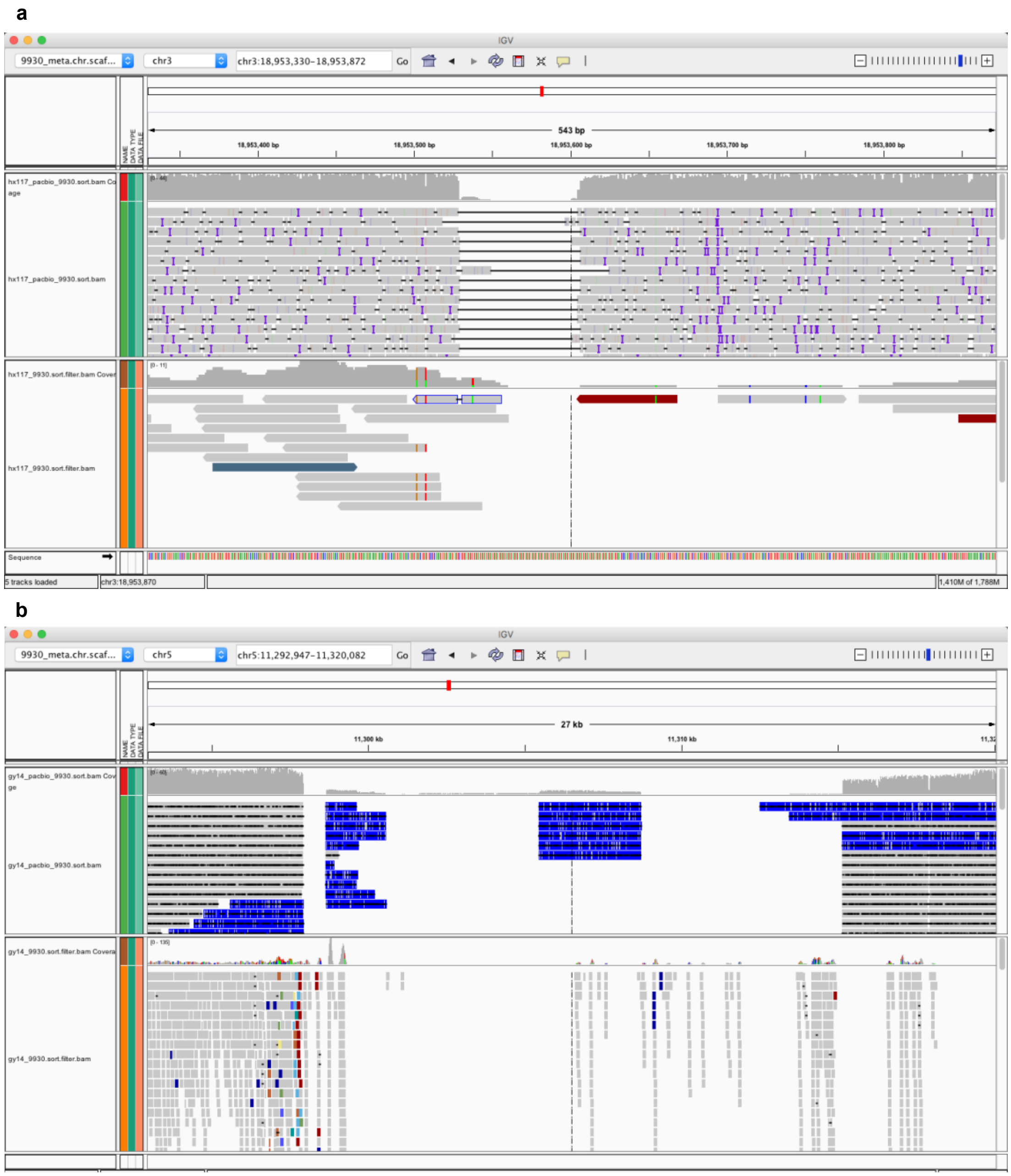

Supplementary Fig. 6. Examples of SVs identified using long reads but not short reads. a, A 77-bp deletion in Hx117. **b,** A 13,852-bp deletion in Gy14. Mapping of PacBio and Illumina reads to the 9930 genome are shown in upper and lower panels, respectively. These two variants were not identified using short reads probably due to the poor mapping around the breakpoints.

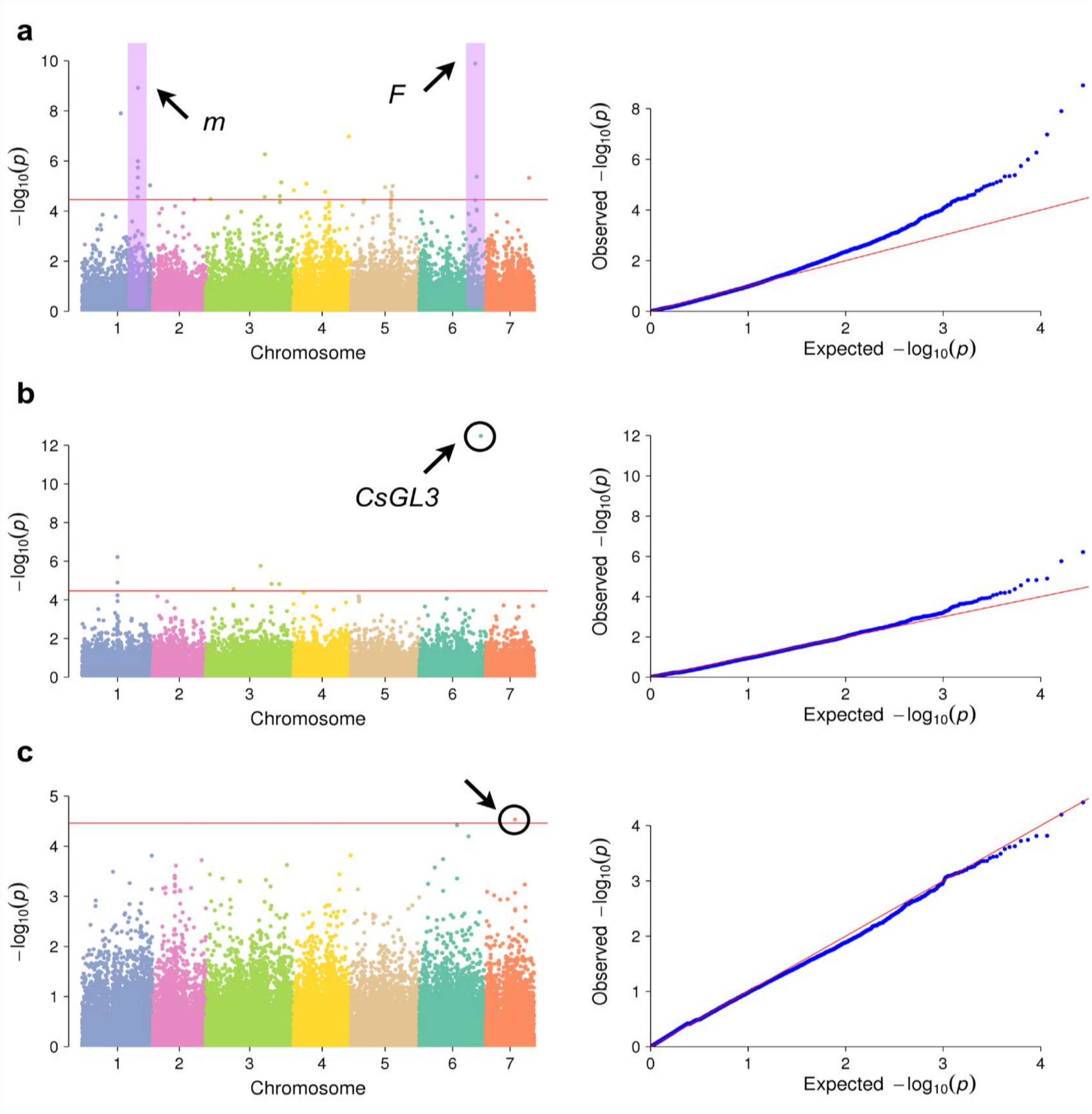

Supplementary Fig. 7. Manhattan and quantile-quantile (QQ) plots of SV-based GWAS of female flower rate on primary branch (a), fruit spine/wart density (b) and branch number (c). The expected *p* values in the QQ plots comply with a uniform distribution.

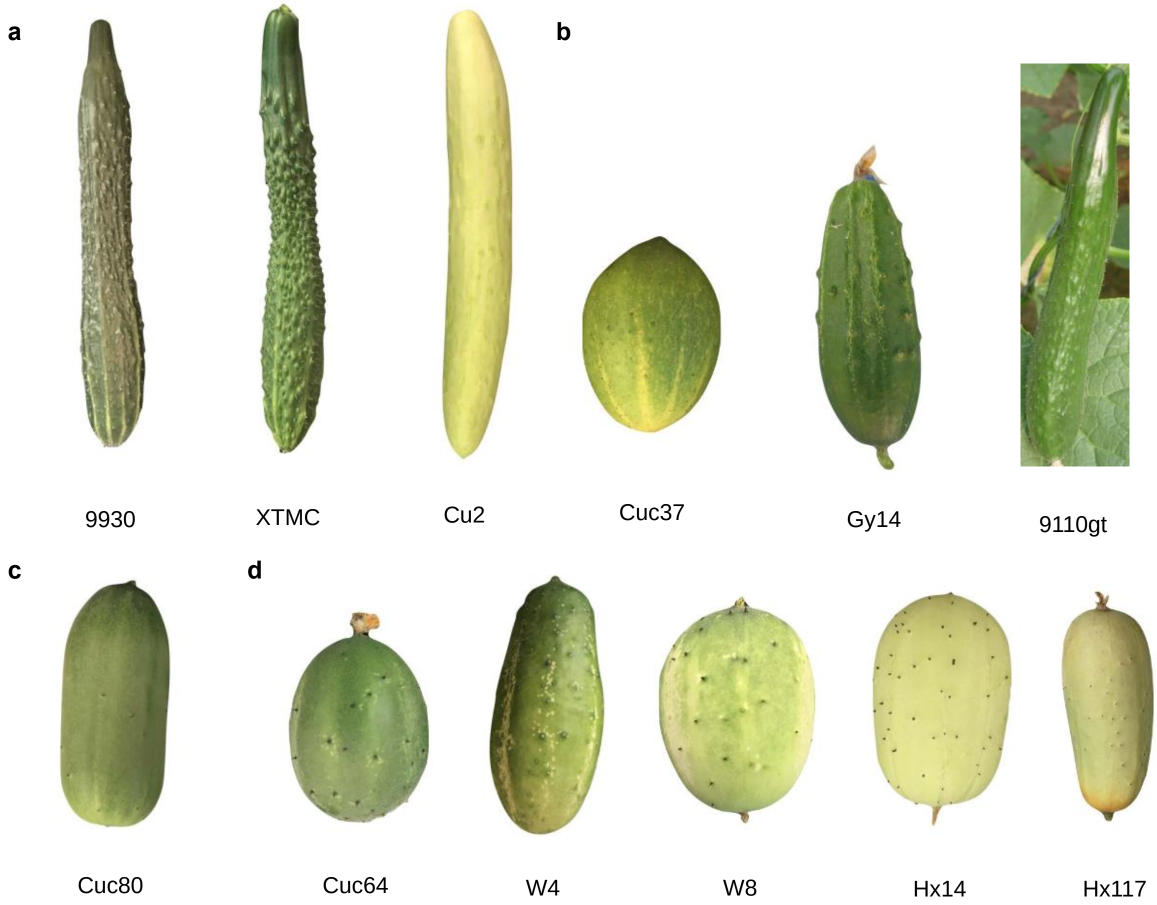

Supplementary Fig. 8. Morphological diversity of cucumber fruit spines and warts among the 12 accessions used in this study. a, Accessions from East-Asian. **b**, Accessions from Eurasian. **c,** Accession from Xishuangbanna. **d**, Accessions from Indian.

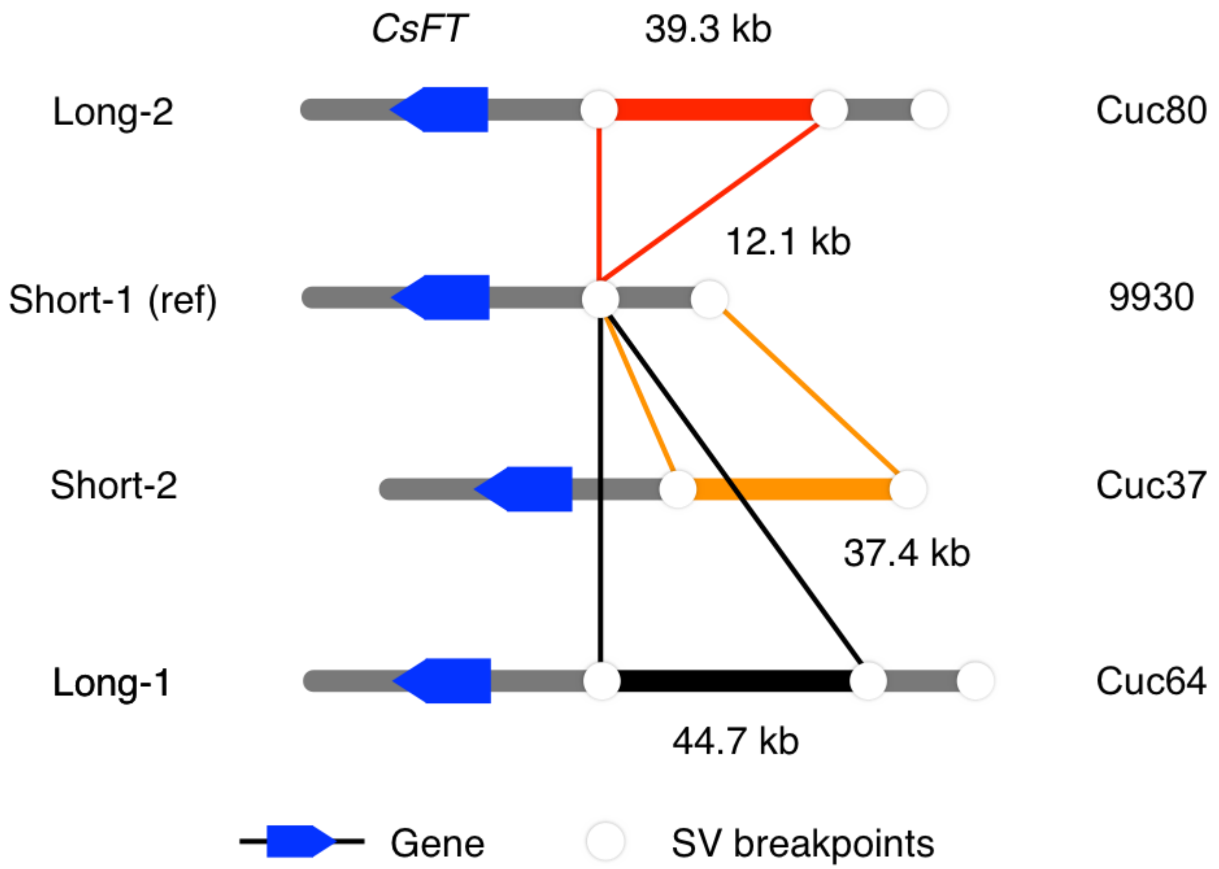

Supplementary Fig. 9. Schematic illustration of alignments of the four types of *CsFT* upstream regions.

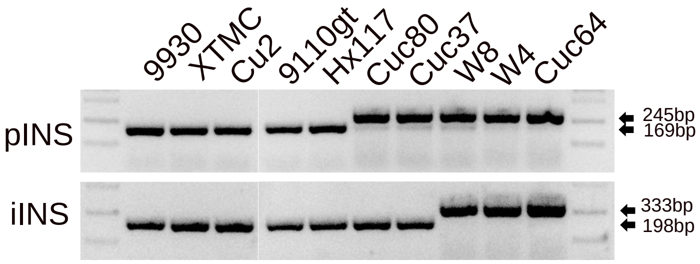

Supplementary Fig. 10. PCR validation of pINS and iINS in 10 accessions. Each experiment is performed once. Source data are provided as a Source Data file.

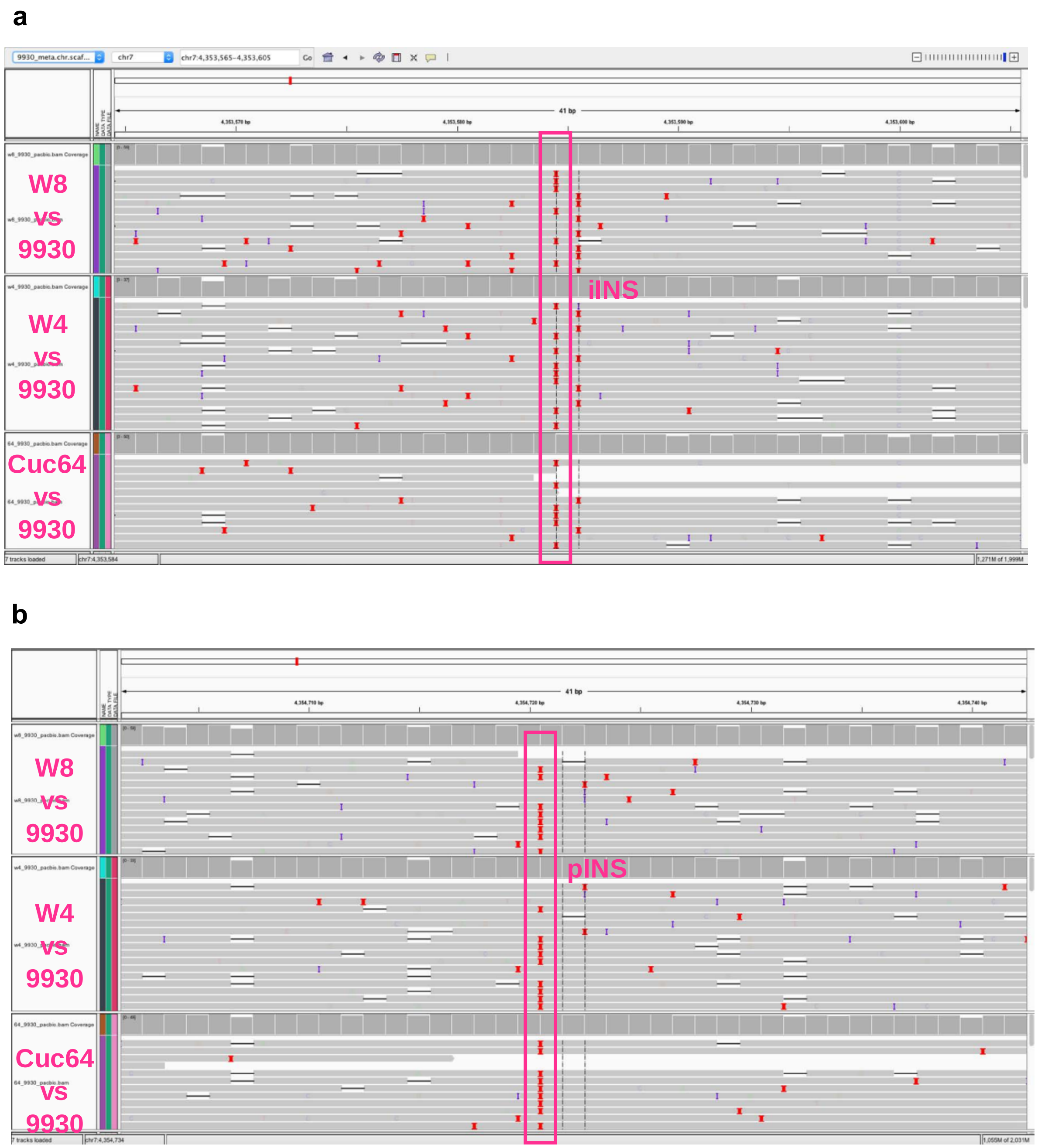

Supplementary Fig. 11. PacBio read mapping supports iINS and pINS. a, Long-read mapping of iINS flanking region. **b,** Long-read mapping of pINS flanking region. The 9930 genome was used as the reference for mapping.

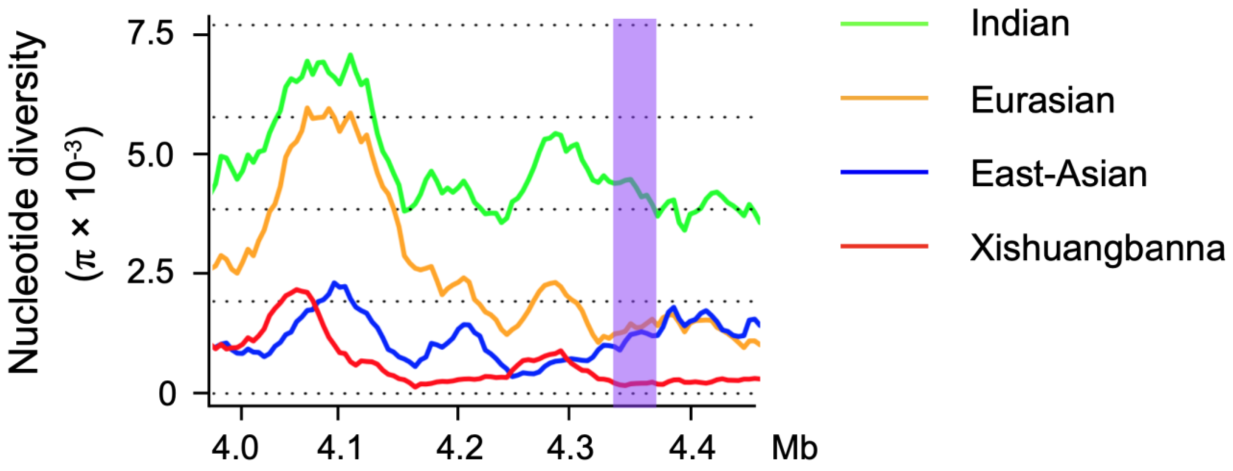

Supplementary Fig. 12. Nucleotide diversity (π) in the *PELPK7.1* and *PELPK7.2* genome region (marked by purple bar). π was calculated based on 100-kb windows.

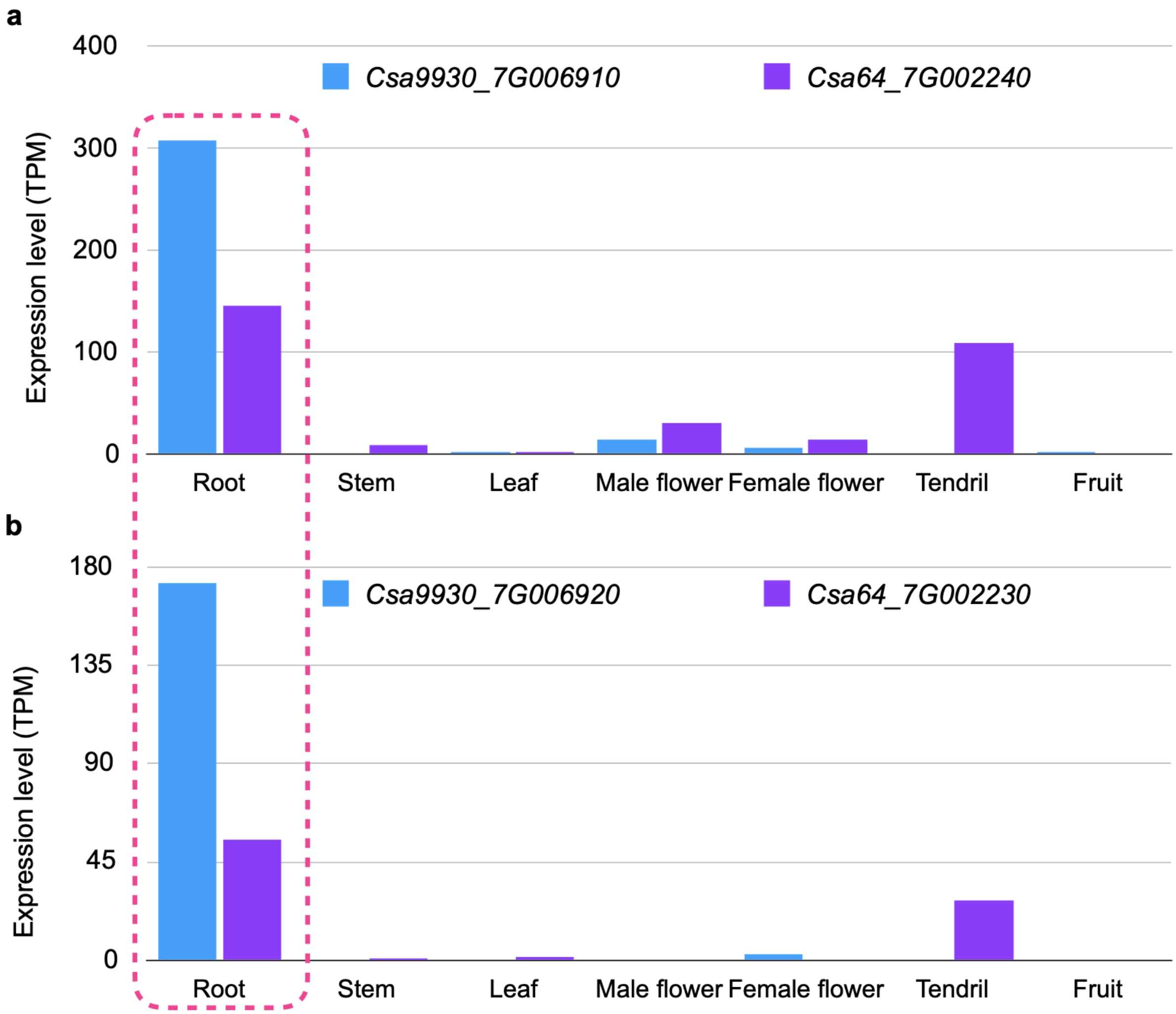

Supplementary Fig. 13. Expression levels of *PELPK7.1* and *PELPK7.2* in cultivated cucumber 9930 and wild cucumber Cuc64. a, Expression of *PELPK7.1* (*Csa9930_7G006910* in 9930 and *Csa64_7G002240* in Cuc64) in seven different tissues. **b,** Expression of *PELPK7.2* (*Csa9930_7G006920* in 9930 and *Csa64_7G002230* in Cuc64) in seven different tissues. TPM, transcripts per million. Source data are provided as a Source Data file.

Supplementary Table 1. Modified Rogers distance (*MR*), Cavalli-Sforza and Edwards distance (*CE*), Shannon's diversity index (*SH*) and genetic coverage value (*CV*) of the 12 accessions and the cucumber core collection of 115 lines. Twenty independent runs were performed, each with 10,000 randomly selected SNPs.

Run	*MR* (12)	*MR* (115)	*CE* (12)	*CE* (115)	*SH* (12)	*SH* (115)	*CV**
1	0.497	0.380	0.500	0.385	9.446	9.512	83.44%
2	0.496	0.378	0.499	0.382	9.446	9.510	83.72%
3	0.492	0.377	0.496	0.381	9.444	9.510	83.71%
4	0.497	0.378	0.500	0.383	9.442	9.511	83.63%
5	0.495	0.379	0.498	0.384	9.446	9.513	83.47%
6	0.498	0.380	0.502	0.384	9.449	9.513	83.45%
7	0.496	0.376	0.499	0.381	9.431	9.508	83.72%
8	0.495	0.377	0.498	0.382	9.438	9.511	83.78%
9	0.498	0.380	0.501	0.385	9.450	9.513	83.36%
10	0.495	0.377	0.499	0.382	9.440	9.510	83.68%
11	0.494	0.378	0.498	0.382	9.445	9.511	83.80%
12	0.496	0.378	0.499	0.383	9.439	9.510	83.50%
13	0.495	0.378	0.498	0.383	9.441	9.511	83.62%
14	0.493	0.375	0.496	0.379	9.444	9.507	83.65%
15	0.495	0.377	0.499	0.381	9.442	9.510	83.79%
16	0.494	0.374	0.497	0.379	9.434	9.508	83.46%
17	0.496	0.377	0.499	0.381	9.436	9.509	83.64%
18	0.495	0.377	0.499	0.381	9.439	9.509	83.74%
19	0.492	0.374	0.496	0.379	9.439	9.506	83.89%
20	0.495	0.377	0.498	0.382	9.442	9.510	83.63%

**CV* represents the proportion of the genetic diversity of the 115-line collection captured by the 12 cucumber accessions

Supplementary Table 2. Summary of PacBio sequencing data.

Accession	Total bases	No. of reads	N50 read length (bp)	Mean read length (bp)
XTMC	18,411,253,399	1,467,719	19,822	12,544
Cu2	22,448,870,253	2,099,019	17,248	10,695
Cuc37	18,898,479,934	2,297,733	12,471	8,225
Gy14	16,541,226,126	1,229,870	20,797	13,450
9110gt	20,286,874,980	2,096,506	15,709	9,677
Cuc80	16,547,985,677	1,744,625	13,264	9,485
Cuc64	15,945,257,333	1,913,418	12,874	8,333
W4	20,249,173,103	1,888,850	17,282	10,720
W8	19,532,540,352	1,986,554	16,284	9,832
Hx14	18,354,892,791	1,479,661	19,120	12,405
Hx117	17,272,792,707	1,532,235	18,037	11,273

Supplementary Table 3. Summary of Illumina resequencing data used in this study.

Accession	Total bases	No. of reads	Read length (bp)
XTMC	5,538,174,000	55,381,740	100
Cu2	5,708,862,300	38,059,082	150
Cuc37	3,735,863,280	41,509,592	90
Gy14	5,816,384,400	58,163,844	100
9110gt	4,904,735,000	49,047,350	100
Cuc80	5,537,630,520	61,529,228	90
Cuc64	4,330,595,850	57,741,278	75
W4	5,525,343,900	61,392,710	90
W8	5,519,869,380	61,331,882	90
Hx14	5,205,132,900	57,834,810	90
Hx117	4,583,354,220	50,926,158	90

Supplementary Table 4. Summary of 10X Genomics sequencing data.

Accession	Total bases	No. of reads	Read length (bp)
Cuc37	28,939,107,000	192,927,380	150
Cuc80	21,762,913,200	145,086,088	150
Cuc64	29,007,008,400	193,380,056	150

Supplementary Table 5. Summary of Hi-C sequencing data.

Accession	Repeat	Total bases	No. of reads	Read length (bp)
Cuc37	Repeat 1	35,597,584,800	237,317,232	150
	Repeat 2	32,818,537,800	218,790,252	150
Cuc80	Repeat 1	34,049,121,900	226,994,146	150
	Repeat 2	32,916,737,700	219,444,918	150
Cuc64	Repeat 1	36,401,990,700	242,679,938	150
	Repeat 2	39,268,682,400	261,791,216	150

Supplementary Table 6. Genome assembly statistics of the 11 cucumber accessions.

	**XTMC**	**Cu2**	**Cuc37**	**Gy14**	**9110gt**	**Cuc80**	**Cuc64**	**W4**	**W8**	**Hx14**	**Hx117**
Assembly length (Mb)	240.0	247.1	238.4	239.4	242.9	237.4	232.5	251.1	241.9	234.6	243.7
No. of contigs	926	851	967	926	830	923	842	894	907	865	1,015
Contig N50[#] (Mb)	2.1	5.3	3.8	2.1	3.9	4.3	4.4	4.7	4.3	1.7	2.1
Longest contig (Mb)	7.7	13.3	18.8	11.2	14.0	10.1	13.5	15.5	18.1	6.8	14.0
No. of scaffolds	/	/	865	/	/	887	796	/	/	/	/
Scaffold N50 (Mb)	/	/	7.9	/	/	7.3	7.9	/	/	/	/
Longest scaffold (Mb)	/	/	18.8	/	/	18.0	16.3	/	/	/	/
No. of super-scaffolds	/	/	238	/	/	290	496	/	/	/	/
Super-scaffold N50 (Mb)	/	/	33.7	/	/	33.3	31.0	/	/	/	/
Longest super-scaffold (Mb)	/	/	43.8	/	/	41.2	41.9	/	/	/	/

[#]N50 refers to the size above which half of the total length of the sequence set can be found

Supplementary Table 7. Summary of annotated transposable elements in the 12 cucumber genomes.

	SINEs (bp / %)	LINEs (bp / %)	LTR elements (bp / %)	DNA elements (bp / %)	Unclassified (bp / %)
9930	0 / 0.00	3,597,475 / 1.59	32,258,069 / 14.26	3,375,240 / 1.49	34,178,558 / 15.11
XTMC	0 / 0.00	5,071,294 / 2.11	40,412,410 / 16.83	3,471,461 / 1.45	40,399,646 / 16.82
Cu2	0 / 0.00	5,294,324 / 2.14	42,005,126 / 17.00	3,514,788 / 1.42	42,359,428 / 17.14
Cuc37	0 / 0.00	4,482,047 / 1.88	42,836,751 / 17.97	3,065,727 / 1.29	38,307,665 / 16.07
Gy14	313,205 / 0.13	7,223,114 / 3.02	41,003,657 / 17.13	8,208,611 / 3.43	27,501,561 / 11.49
9110gt	5,927 / 0.00	5,887,444 / 2.42	46,834,203 / 19.28	3,676,953 / 1.51	37,208,350 / 15.32
Cuc80	7,434 / 0.00	4,387,181 / 1.85	43,095,625 / 18.15	3,006,786 / 1.27	35,150,012 / 14.80
Cuc64	0 / 0.00	5,260,869 / 2.26	40,131,219 / 17.26	2,931,443 / 1.26	33,944,605 / 14.61
W4	0 / 0.00	3,711,032 / 1.48	48,401,617 / 19.28	3,478,371 / 1.39	39,907,171 / 15.89
W8	0 / 0.00	4,702,762 / 1.94	41,481,736 / 17.15	3,344,878 / 1.38	39,255,675 / 16.23
Hx14	0 / 0.00	4,309,772 / 1.84	38,772,670 / 16.53	2,815,615 / 1.20	39,374,747 / 16.79
HX117	0 / 0.00	4,805,179 / 1.97	40,900,204 / 16.78	3,442,478 / 1.41	39,457,439 / 16.19

Supplementary Table 8. Summary of identified LTR-RTs in the 12 cucumber genomes.

Accession	No. of intact LTR-RTs	Length of *Copia* LTR-RTs (bp)	Length of *Gypsy* LTR-RTs (bp)	Length of Unknown LTR-RTs (bp)	Total length (bp)
9930	324	7,039,013	15,978,958	9,240,098	32,258,069
XTMC	473	10,363,287	18,090,814	11,958,309	40,412,410
Cu2	468	6,303,924	23,519,221	12,181,981	42,005,126
Cuc37	497	6,546,061	18,252,751	18,037,939	42,836,751
Gy14	438	7,704,608	17,094,549	16,204,500	41,003,657
9110gt	531	11,914,750	19,548,266	15,371,187	46,834,203
Cuc80	462	5,132,925	19,108,484	18,854,216	43,095,625
Cuc64	440	7,268,350	18,101,476	14,761,393	40,131,219
W4	437	8,183,991	23,023,837	17,193,789	48,401,617
W8	443	6,425,991	19,193,227	15,862,518	41,481,736
Hx14	348	7,751,599	18,816,970	12,204,101	38,772,670
HX117	456	7,629,266	20,355,692	12,915,246	40,900,204

Supplementary Table 9. Statistics of predicted protein-coding genes in the 11 cucumber genomes.

	No. of predicted genes	Average gene length (bp)	Average CDS length (bp)	No. of exons per gene	Average intergenic space distance (bp)	Average intron length (bp)
XTMC	25,167	3,182	1,091	5	5,672	539
Cu2	25,382	3,244	1,109	5	5,747	546
Cuc37	24,490	3,302	1,122	5	6,306	547
Gy14	25,042	3,182	1,091	5	5,580	535
9110gt	24,992	3,256	1,099	5	5,663	549
Cuc80	24,578	3,285	1,123	5	6,225	540
Cuc64	24,583	3,276	1,124	5	5,838	539
W4	25,703	3,270	1,099	5	5,769	554
W8	25,531	3,243	1,095	5	5,538	548
Hx14	24,914	3,234	1,103	5	5,598	544
Hx117	26,033	3,199	1,075	5	5,656	546

Supplementary Table 10. Summary of the seven chromosomal rearrangements between Cuc64 and 9930.

Inversion name	Cuc64			9930		
	Chromosome	Start	End	Chromosome	Start	End
INV4.1	chr4	1	10,745,877	chr4	1	10,690,618
INV4.2	chr4	17,883,271	19,208,399	chr4	18,639,193	19,916,753
INV4.3	chr4	19,228,227	25,957,224	chr4	19,952,352	26,585,698
INV5.1	chr5	1	4,125,964	chr5	1	4,090,476
INV5.2	chr5	4,132,900	13,356,663	chr5	4,090,473	14,380,594
INV5.3	chr5	13,369,466	21,637,222	chr5	13,545,813	22,905,740
INV7.1	chr7	1	6,020,425	chr7	1	5,852,708

Supplementary Table 11. Non-redundant cucumber pan-genome based on protein-coding genes.

Accession	No. of core genes	No. of dispensable genes	No. of total genes (used for clustering)	Core/pan ratio (%)
9930	19,833	4,436	24,269	81.72
XTMC	20,132	4,557	24,689	81.54
Cu2	20,239	4,598	24,837	81.49
Cuc37	19,917	4,151	24,068	82.75
Gy14	20,202	4,450	24,652	81.95
9110gt	20,087	4,489	24,576	81.73
Cuc80	19,950	4,127	24,077	82.86
Cuc64	20,018	4,122	24,140	82.92
W4	20,576	4,557	25,133	81.87
W8	20,601	4,510	25,111	82.04
Hx14	20,012	4,458	24,470	81.78
Hx117	20,862	4,692	25,554	81.64
Pan-genome	18,651	8,171	26,822	69.54

Supplementary Table 12. Top 15 gene ontology (GO) biological process terms enriched in the core genes.

GO ID	Description	Frequency in core genes	Frequency in pan genes	FDR
GO:0009987	cellular process	4498/7,206 (62.42%)	5058/8,265 (61.2%)	2.20E-07
GO:0043412	macromolecule modification	1168/7,206 (16.21%)	1276/8,265 (15.44%)	4.15E-06
GO:0006464	cellular protein modification process	1105/7,206 (15.33%)	1209/8,265 (14.63%)	1.20E-05
GO:0036211	protein modification process	1105/7,206 (15.33%)	1209/8,265 (14.63%)	1.20E-05
GO:0044237	cellular metabolic process	3730/7,206 (51.76%)	4202/8,265 (50.84%)	1.42E-04
GO:0070085	glycosylation	77/7,206 (1.07%)	77/8,265 (0.93%)	4.17E-04
GO:0006486	protein glycosylation	72/7,206 (1%)	72/8,265 (0.87%)	4.90E-04
GO:0009100	glycoprotein metabolic process	72/7,206 (1%)	72/8,265 (0.87%)	4.90E-04
GO:0009101	glycoprotein biosynthetic process	72/7,206 (1%)	72/8,265 (0.87%)	4.90E-04
GO:0043413	macromolecule glycosylation	72/7,206 (1%)	72/8,265 (0.87%)	4.90E-04
GO:0006793	phosphorus metabolic process	1098/7,206 (15.24%)	1214/8,265 (14.69%)	8.27E-04
GO:0006796	phosphate-containing compound metabolic process	1091/7,206 (15.14%)	1207/8,265 (14.6%)	1.00E-03
GO:0044763	single-organism cellular process	1501/7,206 (20.83%)	1672/8,265 (20.23%)	1.31E-03
GO:0009058	biosynthetic process	1883/7,206 (26.13%)	2107/8,265 (25.49%)	1.71E-03
GO:0070647	protein modification by small protein conjugation or removal	118/7,206 (1.64%)	122/8,265 (1.48%)	1.73E-03

Supplementary Table 13. Top 15 gene ontology (GO) biological process terms enriched in the dispensable genes.

GO ID	Description	Frequency in dispensable genes	Frequency in pan genes	FDR
GO:0015074	DNA integration	30/1,059 (2.83%)	38/8,265 (0.46%)	2.10E-18
GO:0009733	response to auxin	32/1,059 (3.02%)	64/8,265 (0.77%)	2.75E-11
GO:0010033	response to organic substance	34/1,059 (3.21%)	105/8,265 (1.27%)	3.20E-06
GO:0009719	response to endogenous stimulus	33/1,059 (3.12%)	101/8,265 (1.22%)	3.20E-06
GO:0009725	response to hormone	33/1,059 (3.12%)	101/8,265 (1.22%)	3.20E-06
GO:0006259	DNA metabolic process	61/1,059 (5.76%)	254/8,265 (3.07%)	8.67E-06
GO:0042221	response to chemical	42/1,059 (3.97%)	163/8,265 (1.97%)	6.86E-05
GO:0000723	telomere maintenance	9/1,059 (0.85%)	14/8,265 (0.17%)	9.90E-05
GO:0032200	telomere organization	9/1,059 (0.85%)	14/8,265 (0.17%)	9.90E-05
GO:0060249	anatomical structure homeostasis	9/1,059 (0.85%)	14/8,265 (0.17%)	9.90E-05
GO:0009611	response to wounding	9/1,059 (0.85%)	19/8,265 (0.23%)	2.27E-03
GO:0050896	response to stimulus	136/1,059 (12.84%)	822/8,265 (9.95%)	5.33E-03
GO:0000280	nuclear division	10/1,059 (0.94%)	29/8,265 (0.35%)	1.76E-02
GO:0008283	cell proliferation	5/1,059 (0.47%)	9/8,265 (0.11%)	1.97E-02
GO:0007127	meiosis I	4/1,059 (0.38%)	6/8,265 (0.07%)	2.16E-02

Supplementary Table 14. Summary of identified SNPs and small InDels.

Accession	No. of SNPs	No. of InDels	Total length of InDels (bp)	Average length of InDels (bp)
XTMC	159,808	121,262	291,229	2.4
Cu2	298,628	132,407	472,752	3.6
Cuc37	531,202	210,330	779,692	3.7
Gy14	480,423	185,738	699,778	3.8
9110gt	317,456	175,862	519,896	3.0
Cuc80	624,986	198,819	860,635	4.3
Cuc64	1,358,519	414,589	1,879,261	4.5
W4	793,213	276,587	1,145,344	4.1
W8	1,371,284	432,819	1,927,486	4.5
Hx14	701,739	269,252	1,022,211	3.8
Hx117	630,902	265,914	945,799	3.6
Total	2,902,954	1,388,197	5,649,184	4.1

Supplementary Table 15. Functional impact of identified SNPs.

Accession	Intergenic	Intron	CDS	Synonymous	Splicing	Stop gained/lost
XTMC	124,167	28,676	7,045	3,767	529	79
Cu2	226,266	57,651	14,836	8,049	1,164	148
Cuc37	408,133	98,635	24,552	13,732	1,982	207
Gy14	365,750	91,737	22,927	12,916	1,834	177
9110gt	241,964	60,653	14,938	8,212	1,194	116
Cuc80	473,961	121,302	29,924	16,808	2,349	230
Cuc64	1,024,242	266,962	67,680	38,255	5,476	517
W4	597,854	156,573	39,107	22,176	3,177	297
W8	956,246	286,806	68,695	39,147	6,243	478
Hx14	532,744	135,384	33,717	19,090	2,693	273
Hx117	479,672	121,386	30,029	16,928	2,402	223

Supplementary Table 16. Functional impact of identified small InDels.

Accession	Intergenic	Intron	CDS	Frameshift	Splicing	Stop gained/lost
XTMC	98,967	20,198	2,169	1,993	349	52
Cu2	108,004	22,688	1,854	1,415	395	22
Cuc37	173,494	34,952	2,015	1,397	589	47
Gy14	148,616	34,170	3,024	2,441	610	55
9110gt	143,705	29,440	2,826	2,424	541	52
Cuc80	161,958	35,077	1,889	1,140	619	42
Cuc64	337,519	73,655	3,678	2,048	1,250	105
W4	223,678	49,625	3,549	2,603	920	69
W8	318,785	84,312	5,367	3,673	1,740	145
Hx14	220,694	45,734	2,951	2,157	791	72
Hx117	211,660	48,947	5,462	4,706	903	88

Supplementary Table 17. Summary of identified canonical insertions.

Accession	No. of SVs	Total length (bp)	Average length (bp)	Max length (bp)	No. of SVs in coding regions	No. of SV-impacted genes	No. of SVs in domestication sweeps
XTMC	1,096	2,652,489	2,420	83,374	27	27	24
Cu2	1,664	2,910,918	1,749	62,006	36	34	26
Cuc37	3,004	4,050,490	1,348	63,426	54	52	43
Gy14	2,504	3,017,529	1,205	52,071	39	39	33
9110gt	1,797	2,552,245	1,420	41,500	30	29	29
Cuc80	3,524	4,032,632	1,144	44,595	61	56	36
Cuc64	7,296	6,666,180	914	74,908	94	92	490
W4	4,387	4,849,062	1,105	51,319	71	66	280
W8	7,577	7,183,410	948	62,932	95	95	531
Hx14	3,601	3,921,620	1,089	56,862	62	55	124
Hx117	3,331	3,997,964	1,200	53,205	60	56	105
Total	17,130	24,816,009	1,449	83,374	333	291	940

Supplementary Table 18. Summary of identified canonical deletions.

Accession	No. of SVs	Total length (bp)	Average length (bp)	Max length (bp)	No. of SVs in coding regions	No. of SV-impacted genes	No. of SVs in domestication sweeps
XTMC	1,231	2,054,473	1,669	136,303	63	121	28
Cu2	2,167	2,852,805	1,316	136,315	121	168	44
Cuc37	3,154	3,305,045	1,048	130,439	132	173	56
Gy14	2,964	3,531,394	1,191	136,303	126	175	53
9110gt	2,096	2,557,241	1,220	135,907	108	167	48
Cuc80	3,458	3,634,360	1,051	68,158	158	200	33
Cuc64	6,702	5,846,932	872	46,048	288	360	478
W4	4,667	4,336,096	929	136,303	209	269	316
W8	7,284	6,056,794	832	39,892	282	358	516
Hx14	4,153	3,761,191	906	137,097	161	213	210
Hx117	3,844	3,829,068	996	135,907	183	248	144
Total	19,334	13,398,747	693	137,097	1,012	930	1,143

Supplementary Table 19. Summary of identified complex insertions.

Accession	No. of SVs	Total length (bp)	Average length (bp)	Max length (bp)	No. of SVs in coding regions	No. of SV-impacted genes	No. of SVs in domestication sweeps
XTMC	466	941,180	2,020	36,781	3	3	0
Cu2	878	1,923,063	2,190	124,097	6	5	1
Cuc37	1,350	1,582,073	1,172	40,947	8	12	1
Gy14	1,087	1,372,550	1,263	53,035	13	10	1
9110gt	795	996,796	1,254	25,349	5	9	1
Cuc80	1,520	1,612,252	1,061	48,778	7	5	1
Cuc64	3,708	3,683,408	993	72,828	14	11	15
W4	2,030	2,137,196	1,053	36,855	11	8	14
W8	3,707	4,020,383	1,085	72,828	10	8	25
Hx14	1,717	2,390,015	1,392	86,501	7	6	4
Hx117	1,544	2,313,902	1,499	87,193	7	8	5
Total	9,399	9,673,969	1,029	124,097	78	63	46

Supplementary Table 20. Summary of identified complex deletions.

Accession	No. of SVs	Total length (bp)	Average length (bp)	Max length (bp)	No. of SVs in coding regions	No. of SV-impacted genes	No. of SVs in domestication sweeps
XTMC	417	615,267	1,475	39,889	43	43	3
Cu2	819	1,104,607	1,349	86,970	58	88	6
Cuc37	1,130	1,198,757	1,061	89,618	73	99	2
Gy14	912	686,441	753	15,699	51	51	4
9110gt	722	637,701	883	24,333	43	46	2
Cuc80	1,276	1,016,786	797	46,430	73	82	3
Cuc64	2,745	2,522,027	919	82,614	176	212	182
W4	1,654	1,646,538	995	60,231	128	131	114
W8	2,690	2,053,505	763	32,483	162	165	163
Hx14	1,403	1,063,863	758	23,815	81	89	61
Hx117	1,246	862,205	692	15,699	73	71	33
Total	8,049	7,627,939	948	89,618	634	600	390

Supplementary Table 21. Summary of identified inversions.

Accession	No. of SVs	Total length (bp)	Average length (bp)	Max length (bp)	No. of SVs in coding regions	No. of SV-impacted genes	No. of SVs in domestication sweeps
XTMC	9	629,759	69,973	291,854	7	58	0
Cu2	20	721,223	36,061	239,883	12	78	0
Cuc37	21	664,972	31,665	239,883	10	60	0
Gy14	22	723,449	32,884	241,786	9	71	0
9110gt	13	321,468	24,728	258,396	3	27	0
Cuc80	39	966,556	24,783	249,794	16	73	0
Cuc64	40	1,415,520	35,388	435,827	26	155	3
W4	37	1,332,367	36,010	347,532	21	145	2
W8	42	1,005,309	23,936	478,382	19	107	4
Hx14	35	1,682,476	48,071	454,038	19	164	1
Hx117	21	926,075	44,099	239,883	11	88	2
Total	196	5,469,389	27,905	478,382	109	521	9

Supplementary Table 22. Summary of identified intra-chromosomal translocations.

Accession	No. of SVs	Total length (bp)	Average length (bp)	Max length (bp)	No. of SVs in coding regions	No. of SV-impacted genes	No. of SVs in domestication sweeps
XTMC	29	529,688	18,265	150,053	7	27	0
Cu2	66	937,882	14,210	233,246	14	79	2
Cuc37	71	1,222,129	17,213	282,061	23	101	1
Gy14	66	1,799,469	27,265	709,697	19	139	5
9110gt	42	741,868	17,664	204,724	14	58	0
Cuc80	98	1,531,185	15,624	182,755	40	93	0
Cuc64	104	753,960	7,250	77,356	22	57	1
W4	85	2,069,984	24,353	319,737	35	212	5
W8	125	2,531,322	20,251	816,765	30	190	2
Hx14	61	1,660,993	27,229	282,061	23	143	2
Hx117	52	1,317,101	25,329	250,449	17	84	0
Total	702	9,694,700	13,810	816,765	226	775	17

Supplementary Table 23. Summary of identified inter-chromosomal translocations.

Accession	No. of SVs	Total length (bp)	Average length (bp)	Max length (bp)	No. of SVs in coding regions	No. of SV-impacted genes	No. of SVs in domestication sweeps
XTMC	34	622,298	18,303	283,468	7	53	1
Cu2	106	1,179,689	11,129	205,505	17	46	4
Cuc37	213	2,634,411	12,368	255,733	47	99	5
Gy14	130	1,155,519	8,889	283,468	20	70	2
9110gt	90	1,213,231	13,480	230,797	20	102	2
Cuc80	280	3,480,956	12,432	257,407	48	87	5
Cuc64	204	1,310,580	6,424	239,431	27	74	7
W4	191	1,309,654	6,857	251,410	29	82	4
W8	242	1,351,703	5,586	243,658	26	79	6
Hx14	144	1,714,879	11,909	221,866	33	93	3
Hx117	161	1,133,605	7,041	72,643	27	53	6
Total	1,404	7,043,821	5,017	283,468	232	257	33

Supplementary Table 24. Number of canonical deletions detected in the 11 cucumber accessions that were supported by Illumina reads.

Accession	No. of all canonical deletions	No. of canonical deletions supported by Illumina reads	Percentage (%)
XTMC	1,231	858	69.69
Cu2	2,167	1,572	72.54
Cuc37	3,154	2,751	87.22
Gy14	2,964	2,443	82.42
9110gt	2,096	1,643	78.38
Cuc80	3,458	3,197	92.45
Cuc64	6,702	5,373	80.17
W4	4,667	3,552	76.10
W8	7,284	6,230	85.52
Hx14	4,153	3,545	85.35
Total	3,844	2,914	75.80

Supplementary Table 25. Number of canonical insertions detected in the 11 cucumber accessions that were supported by Illumina reads.

Accession	No. of all canonical insertions	No. of canonical insertions supported by Illumina reads	Percentage (%)
XTMC	1,097	982	89.52
Cu2	1,665	1,562	93.81
Cuc37	3,005	2,815	93.68
Gy14	2,505	2,344	93.57
9110gt	1,798	1,628	90.55
Cuc80	3,525	3,339	94.72
Cuc64	7,297	7,014	96.12
W4	4,388	4,184	95.35
W8	7,578	7,263	95.84
Hx14	3,602	3,393	94.20
Total	3,332	3,134	94.06

Supplementary Table 26. Number of complex deletions detected in the 11 cucumber accessions that were supported by Illumina reads.

Accession	No. of all complex deletions	No. of complex deletions supported by Illumina reads	Percentage (%)
XTMC	417	301	72.18
Cu2	819	515	62.88
Cuc37	1,130	960	84.95
Gy14	912	750	82.23
9110gt	722	579	80.19
Cuc80	1,276	1,096	85.89
Cuc64	2,745	1,983	72.24
W4	1,654	1,194	72.18
W8	2,690	2,232	82.97
Hx14	1,403	1,214	86.52
Hx117	1,246	899	72.15

Supplementary Table 27. Number of complex insertions detected in the 11 cucumber accessions that were supported by Illumina reads.

Accession	No. of all complex insertions	No. of complex insertions supported by Illumina reads	Percentage (%)
XTMC	467	400	85.65
Cu2	879	735	83.62
Cuc37	1,351	1,239	91.71
Gy14	1,088	957	87.96
9110gt	796	694	87.19
Cuc80	1,521	1,365	89.74
Cuc64	3,709	3,401	91.70
W4	2,031	1,843	90.74
W8	3,708	3,374	90.99
Hx14	1,718	1,449	84.34
Hx117	1,545	1,374	88.93

Supplementary Table 28. Summary of domestication-associated SVs.

SV type	No. of SVs	Total length (bp)	Average length (bp)	Max length (bp)	No. of SVs in coding regions	No. of SVs in gene promoters
Canonical insertion	940	1,167,441	1,242	31,354	22	260
Canonical deletion	1,143	718,505	629	36,259	60	286
Complex insertion	46	8,746	190	971	1	12
Complex deletion	390	289,326	742	22,816	27	111
Inversion	9	82,234	9,137	45,542	6	6
Translocation	50	1,213,756	24,275	709,697	8	0
Total	2,578	3,480,008	36,215	846,639	124	675

Supplementary Table 29. Summary of SVs displaying potential domestication signals.

SV type	No. of SVs	Total length (bp)	Average length (bp)	Max length (bp)	No. of SVs in coding regions	No. of SVs in gene promoters
Canonical insertion	3,546	2,632,703	742	41,884	59	988
Canonical deletion	2,372	1,447,370	610	29,490	151	612
Complex insertion	1,758	1,636,826	931	40,252	4	39
Complex deletion	966	498,026	516	22,816	67	269
Inversion	9	23,941	2,660	7,615	13	5
Total	8,651	6,238,866	5,459	41,884	294	1,913

Supplementary Table 30. Number of SVs that possibly altered nearby gene expression during cucumber domestication.

SV type	Root	Stem	Leaf	Male flower	Female flower	Fruit	Tendril	At least one tissue
CDS-SV	60	63	51	61	61	64	65	131
Promoter-SV	579	631	459	659	635	657	691	1,480
Total	639	694	510	720	696	721	756	1,611

Supplementary references

1. Qi, J. *et al.* A genomic variation map provides insights into the genetic basis of cucumber domestication and diversity. *Nat. Genet.* **45**, 1510-5 (2013).
2. Tettelin, H. *et al.* Genome analysis of multiple pathogenic isolates of Streptococcus agalactiae: implications for the microbial "pan-genome". *Proc. Natl. Acad. Sci. USA* **102**, 13950-5 (2005).
3. Zhao, Y. *et al.* PanGP: a tool for quickly analyzing bacterial pan-genome profile. *Bioinformatics* **30**, 1297-9 (2014).
