# Supplementary material for: Graph-based pan-genome reveals structural and sequence variations related to agronomic traits and domestication in cucumber
Source: Nat Commun. 2022 Feb 3;13:682. doi: 10.1038/s41467-022-28362-0 (PMC8813957; doi:10.1038/s41467-022-28362-0)
Supplement: Supplementary file 3 — Description of Additional Supplementary Files [file 41467_2022_28362_MOESM3_ESM.pdf]

Description of Additional Supplementary Files

File Name: Supplementary Data 1
Description: Number of members of the 435 LTR-RT family in the 12 cucumber accessions.

File Name: Supplementary Data 2
Description: Matrix of 26,822 nonredundant pan-genes.

File Name: Supplementary Data 3
Description: Information of SVs identified from 12 cucumber genomes.

File Name: Supplementary Data 4
Description: Summary of SVs that affected gene CDS.

File Name: Supplementary Data 5
Description: Summary of identified selective sweeps.

File Name: Supplementary Data 6
Description: Summary of domestication-associated SVs.

File Name: Supplementary Data 7
Description: Summary of potential domestication-associated SVs.

File Name: Supplementary Data 8
Description: Summary of CDS-SVs displaying significantly altered expression of the closest gene in at least one tissue.

File Name: Supplementary Data 9
Description: Summary of promoter-SVs displaying significantly altered expression of the closest in at least one tissue.
