# Supplementary material for: Graph-based pan-genome reveals structural and sequence variations related to agronomic traits and domestication in cucumber
Source: Nat Commun. 2022 Feb 3;13:682. doi: 10.1038/s41467-022-28362-0 (PMC8813957; doi:10.1038/s41467-022-28362-0)
Supplement: Supplementary file 13 — Reporting Summary [file 41467_2022_28362_MOESM13_ESM.pdf]

nature research

Corresponding author(s): Zhonghua Zhang

Last updated by author(s): Jan 7, 2022

Reporting Summary

Nature Research wishes to improve the reproducibility of the work that we publish. This form provides structure for consistency and transparency in reporting. For further information on Nature Research policies, see our Editorial Policies and the Editorial Policy Checklist.

Statistics

For all statistical analyses, confirm that the following items are present in the figure legend, table legend, main text, or Methods section.

n/a	Confirmed	
☐	☒	The exact sample size (*n*) for each experimental group/condition, given as a discrete number and unit of measurement
☐	☒	A statement on whether measurements were taken from distinct samples or whether the same sample was measured repeatedly
☐	☒	The statistical test(s) used AND whether they are one- or two-sided<br>*Only common tests should be described solely by name; describe more complex techniques in the Methods section.*
☒	☐	A description of all covariates tested
☒	☐	A description of any assumptions or corrections, such as tests of normality and adjustment for multiple comparisons
☐	☒	A full description of the statistical parameters including central tendency (e.g. means) or other basic estimates (e.g. regression coefficient) AND variation (e.g. standard deviation) or associated estimates of uncertainty (e.g. confidence intervals)
☐	☒	For null hypothesis testing, the test statistic (e.g. *F*, *t*, *r*) with confidence intervals, effect sizes, degrees of freedom and *P* value noted<br>*Give P values as exact values whenever suitable.*
☒	☐	For Bayesian analysis, information on the choice of priors and Markov chain Monte Carlo settings
☒	☐	For hierarchical and complex designs, identification of the appropriate level for tests and full reporting of outcomes
☒	☐	Estimates of effect sizes (e.g. Cohen's *d*, Pearson's *r*), indicating how they were calculated

Our web collection on statistics for biologists contains articles on many of the points above.

Software and code

Policy information about availability of computer code

Data collection	No software was used to collect data for this study. Sequencing platforms used to generate the raw data are listed as followed: PacBio RS II, PacBio Sequel, Illumina HiSeq X Ten.
Data analysis	Core Hunter v.2.0, GenoCore (no versions, https://github.com/lovemun/genocore), CANU v1.8, BLAST v2.2.30+, Pilon v1.22, ALLMAPS (no versions, https://github.com/tanghaibao/jcvi/wiki/ALLMAPS), ARCS v1.0.2, Juicer v1.6.2, 3D-DNA v180419, HiCPlotter v0.6.6, MUMmer v4.0.0beta2, BUSCO29 v5.2.1, RepeatModeler v1.0.11, LTR_FINDER v1.0.7, LTR_harvest v1.5.10, LTR_retriever v1.6, RepeatMasker v1.332, cd-hit-est v4.8.1, EVidenceModeler v1.1.1, SPALN v2.3.2, InterProScan v5.27-66.0, OrthoMCL v2.0.9, MUSCLE v3.8.31, Gblocks 0.91b, IQ-TREE v1.6.12, GET_HOMOLOGUES-EST pipeline v3.0.9, HISAT2 v2.1.0, StringTie v1.3.4, R v3.6.1, axtChain (v369), chainNet (v369), diffseq (v6.5.7), BWA v0.7.17-r1188, SAMtools v1.3, BCFtools v1.10.2, snpEff v4.3i, BLAT v34x12, LUMPY v0.3.1, DELLY v0.8.7, SyRI v1.0, minimap2 v2.9-r748-dirty, vg v1.23.0, EMMAX v20120205, PLINK v1.90b6.22, Genetic type 1 Error Calculator v0.2, GATK v3.2-2, XP-CLR v1.1 and SynOrth v1.0

For manuscripts utilizing custom algorithms or software that are central to the research but not yet described in published literature, software must be made available to editors and reviewers. We strongly encourage code deposition in a community repository (e.g. GitHub). See the Nature Research guidelines for submitting code & software for further information.

Data

Policy information about availability of data

All manuscripts must include a data availability statement. This statement should provide the following information, where applicable:

- Accession codes, unique identifiers, or web links for publicly available datasets
- A list of figures that have associated raw data
- A description of any restrictions on data availability

Genome assemblies of the 11 cucumber accessions have been deposited in NCBI GenBank under the accession number PRJNA657438 [https://

www.ncbi.nlm.nih.gov/bioproject/?term=PRJNA657438]. Raw PacBio sequencing data of the 11 cucumber accessions and Hi-C sequencing reads of Cuc37, Cuc80 and Cuc64 have been deposited in the NCBI sequence read archive (SRA) under the accession number SRP278022 [https://www.ncbi.nlm.nih.gov/sra/?term=SRP278022].

Field-specific reporting

Please select the one below that is the best fit for your research. If you are not sure, read the appropriate sections before making your selection.

- [x] Life sciences
- [ ] Behavioural & social sciences
- [ ] Ecological, evolutionary & environmental sciences

For a reference copy of the document with all sections, see nature.com/documents/nr-reporting-summary-flat.pdf

Life sciences study design

All studies must disclose on these points even when the disclosure is negative.

Sample size	For pan-genome construction, 12 representative cucumber accessions were used. These accessions were selected based on their phylogenetic relationship and represented genetic diversity in the cucumber germplasm.
Data exclusions	No samples were excluded in this study. Filters applied to eliminate low-quality genetic variants were properly described in the Methods section.
Replication	Three biological replicates were applied in qRT-PCR experiments. All replications were successful and were used.
Randomization	Randomization does not directly apply to the genome sequencing and assembly. To evaluate the representativeness of the 12 accessions selected in this study, we randomly selected 10,000 SNPs with missing genotype rates < 0.1 from a previously genotyped cucumber core collection of 115 lines (3,530,580 SNPs), and repeated this process for 20 times.
Blinding	Blinding does not apply to this study, as the study focuses on comparative genomics and blinding is not necessary.

Reporting for specific materials, systems and methods

We require information from authors about some types of materials, experimental systems and methods used in many studies. Here, indicate whether each material, system or method listed is relevant to your study. If you are not sure if a list item applies to your research, read the appropriate section before selecting a response.

Materials & experimental systems

n/a	Involved in the study	
x		Antibodies
x		Eukaryotic cell lines
x		Palaeontology and archaeology
x		Animals and other organisms
x		Human research participants
x		Clinical data
x		Dual use research of concern

Methods

n/a	Involved in the study	
x		ChIP-seq
x		Flow cytometry
x		MRI-based neuroimaging
