# Supplementary figures and images for: Graph-based pan-genome reveals structural and sequence variations related to agronomic traits and domestication in cucumber
Source: Nat Commun. 2022 Feb 3;13:682. doi: 10.1038/s41467-022-28362-0 (PMC8813957; doi:10.1038/s41467-022-28362-0)

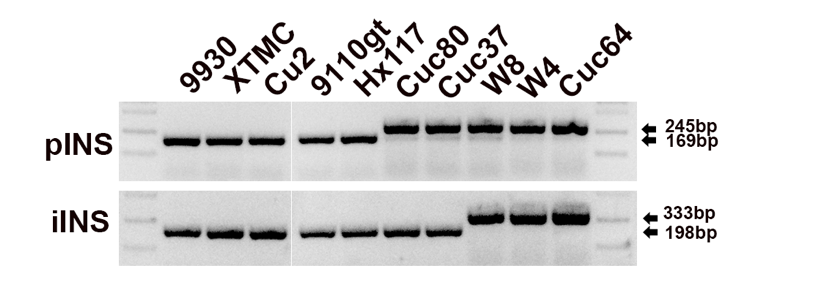


**Source Data 1. The uncropped scan of a gel plot corresponding to Supplementary Fig. 10.**

Supplement: Supplementary file 14 — Source Data [file 41467_2022_28362_MOESM14_ESM.zip › Source Data Fig. for Supp Fig. 10.docx]
